# Risk Factors for Multidrug-Resistant Tuberculosis among Patients with Pulmonary Tuberculosis at the Central Chest Institute of Thailand

**DOI:** 10.1371/journal.pone.0139986

**Published:** 2015-10-07

**Authors:** Charoen Chuchottaworn, Vipa Thanachartwet, Piamlarp Sangsayunh, Thu Zar Myint Than, Duangjai Sahassananda, Manoon Surabotsophon, Varunee Desakorn

**Affiliations:** 1 Division of Respiratory Medicine, Central Chest Institute of Thailand (CCIT), Nonthaburi, Thailand; 2 Department of Clinical Tropical Medicine, Faculty of Tropical Medicine, Mahidol University, Bangkok, Thailand; 3 Information Technology Unit, Faculty of Tropical Medicine, Mahidol University, Bangkok, Thailand; 4 Division of Pulmonary and Critical Care Medicine, Ramkhamhaeng Hospital, Bangkok, Thailand; University of Minnesota, UNITED STATES

## Abstract

There are limited data available on the risk factors for multidrug-resistant tuberculosis (MDR-TB). Therefore, we here conducted a retrospective matched case−control study among adults with pulmonary TB who received treatment at the Central Chest Institute of Thailand (CCIT) between January 2007 and December 2013, in order to determine the risk factors associated with MDR-TB among patients with pulmonary TB. We identified 145 patients with pulmonary MDR-TB (cases) and 145 patients with drug-sensitive pulmonary TB (controls). Multivariate analysis identified the independent risk factors for MDR-TB as follows: (1) ≥ 2 episodes of prior pulmonary TB (odds ratio [OR] 39.72, 95% confidence interval (95% CI) 7.86−200.66), (2) duration of illness > 60 days (OR 3.08, 95% CI 1.52−6.22), (3) sputum acid fast bacilli smear 3+ (OR 13.09, 95% CI 4.64−36.91), (4) presence of lung cavities (OR 3.82, 95% CI 1.89−7.73), and (5) presence of pleural effusion (OR 2.75, 95% CI 1.06−7.16). Prior pulmonary TB management with a non-category I regimen (*P* = 0.012) and having treatment failure or default as treatment outcomes (*P* = 0.036) were observed in a higher proportion among patients with MDR-TB. Particular characteristics of lung cavities, including the maximum diameter ≥ 30 mm (*P* < 0.001), the number of cavities ≥ 3 (*P* = 0.001), bilateral involvement (*P* < 0.001), and ≥ 2 lung zones involved (*P* = 0.001) were more commonly observed in patients with MDR-TB. In conclusion, these clinical factors and chest radiographic findings associated with MDR-TB among patients with pulmonary TB may help physicians to provide proper management of cases for prevention of the development and spread of MDR-TB in future.

## Introduction

Tuberculosis (TB) is caused by *Mycobacterium tuberculosis* and remains one of the leading causes of death worldwide, despite the availability of effective anti-TB drugs [1]. In 2013, the World Health Organization (WHO) reported that approximately one-third of the world’s population was infected with *M*. *tuberculosis*; of which 9.0 million people were estimated to develop TB. The majority of patients with TB (56.0%) were reported from South-East Asia and the Western Pacific, with approximately 1.1 million patients with TB dying due to the disease [2].

During anti-TB treatment, there is selection pressure on a population of *M*. *tuberculosis* resulting in the occurrence of spontaneous resistance-causing mutations in a number of susceptible bacilli, which then gradually increase to become the dominant strain [3]. People who are infected with an already drug-resistant strain could develop primary resistance, which is commonly observed in newly diagnosed TB patients. When resistance mutants arise during treatment with anti-TB drugs, it is considered acquired resistance, which is usually found in previously treated patients [4]. During global surveillance from 1999 to 2002, resistance to any of the four main anti-TB drugs, viz., isoniazid (H, INH), rifampicin (R, RMP), ethambutol (E, EMB), and streptomycin (S, SM) were reported in 10.2% of patients with TB, and these numbers have gradually been increasing [5, 6].

At present, multidrug-resistant TB (MDR-TB), which is defined as drug resistance at least to both INH and RMP has spread globally since 2000 [2,6,7]. In 2013, the WHO reported that approximately 480,000 of the world’s population had MDR-TB, resulting in approximately 210,000 (43.8%) deaths. Approximately 60% of patients with MDR-TB were reported from India, China, and the Russian Federation and it was estimated that 3.5% of newly diagnosed TB patients and 20.5% of previously treated patients had MDR-TB [2].

Recently, it was reported that being human immunodeficiency virus (HIV)-positive was not a risk factor for MDR-TB and that the prevalence of TB has been increasing among patients who are HIV-negative since 2005 [7,8]. During 2006 to 2014, several investigators reported risk factors for MDR-TB and the majority consistently identified previous treatment with an anti-TB drug as one of these risk factors [9–25]. However, a number of reports have explored factors among previously treated patients that are associated with the occurrence of MDR-TB; these included (1) age ≥ 45 years, (2) duration of first anti-TB treatment > 8 months, (3) treatment with INH and RMP > 180 days, (4) absence of fixed dose-combinations, (5) delayed initiation of anti-TB treatment > 60 days, (6) > 3 episodes of anti-TB treatment, and (7) adverse effects of anti-TB treatment [16,18,23,24].

In Thailand, TB is a significant public health problem, although the prevalence has decreased from 161 per 100,000 in the general population in 2011 to 149 per 100,000 in 2013. The majority of TB patients (80.0%) had pulmonary TB with a treatment success rate of 81% among new or relapse cases; MDR-TB developed in 19.0% of previously treated patients, but in only 2.0% of newly diagnosed TB patients [2]. However, the drug-susceptibility testing (DST) as well as the management of patients with MDR-TB could be applied only in some hospitals in Thailand, such as the Provincial Hospitals, University Hospitals, and the Central Chest Institute of Thailand (CCIT). The CCIT in Nonthaburi province was established as the primary and tertiary center for treatment of patients with pulmonary TB, particularly MDR-TB, in Thailand. Thus, the majority of patients with pulmonary MDR-TB were referred to the CCIT for treatment when the physician suspected MDR-TB or when the sputum culture isolates of *M*. *tuberculosis* showed a drug-resistant strain. A recent report from Thailand showed a delay in the results of sputum culture for TB and DST and that only 5.8% of patients with MDR-TB were empirically treated with an appropriate regimen for MDR-TB before the DST results became available. Additionally, 31.3% of patients with MDR-TB received an appropriate regimen after receiving the DST results [26].

In clinical practice, chest radiography is a simple diagnostic tool and usually helps physicians to identify and manage pulmonary TB when the results of culture and DST are not yet available. A previous study showed that pulmonary TB patients with *M*. *tuberculosis* isolated from sputum, who had bilateral lung involvement upon presentation, were more likely to be patients with drug-resistance to any first-line anti-TB drugs than to be patients showing drug-sensitivity to all first-line anti-TB drugs [27]. However, chest radiographic findings of patients with pulmonary MDR-TB, as compared to those with non-MDR-TB, varied among studies due to the differences in the definition of patients with non-MDR-TB [28–30]. The treatment success rate of patients with drug-resistance to one of the first-line anti-TB drugs, particularly INH mono-resistant TB (≥ 90%), was similar to that of patients with TB that was drug-sensitive to all first-line anti-TB drugs [31–33], but the treatment success rate of patients with MDR-TB was only 48% and the mortality rate ranged from 1% to 30% among patients with MDR-TB [34]. Thus, prevention and rapid detection of MDR-TB are priorities for controlling MDR-TB.

No previous report has attempted to identify risk factors from among clinical factors, microbiology data, and chest radiographic findings in pulmonary MDR-TB in Thailand. Therefore, we here conducted a retrospective matched case−control study to determine the risk factors associated with MDR-TB among adults with pulmonary TB.

## Materials and Methods

The Standards for the Reporting of Observational Studies in Epidemiology (STROBE) were followed in this study. The study protocol was approved by the Ethics Committee of the Faculty of Tropical Medicine, Mahidol University, and the Ethics Committee of the CCIT in Thailand. Written informed consent was not obtained as it was specifically waived by the approving Ethics Committee of the Faculty of Tropical Medicine and the CCIT. Data were anonymized before analysis by de-identifying patient data.

### Study design and population

This study was conducted as a retrospective matched case−control study from a prospective cohort of 8424 newly registered pulmonary TB patients who received treatment in the outpatient department of the TB clinic at the CCIT from January 2007 to December 2013. Cases were patients with pulmonary MDR-TB. Pulmonary MDR-TB was defined as sputum culture showing *M*. *tuberculosis* and where DST revealed drug-resistance to both INH and RMP [35]. Controls were pulmonary TB patients with sputum culture showing *M*. *tuberculosis* and where DST revealed drug-sensitivity to all four first-line anti-TB drugs, i.e., INH, RMP, EMB, and SM.

From January 2007 to December 2013, a total of 8424 pulmonary TB patients newly registered at the outpatient department of TB clinic were recorded in the registry books. A total of 5059 pulmonary TB patients had AFB smear-positive sputum and *M*. *tuberculosis* isolated from sputum culture documented in the registry book, comprising 558 patients with pulmonary MDR-TB and 4501 patients with pulmonary non-MDR-TB. Any patients who were identified by searching medical records and microbiology laboratory records of the central laboratory and who met the study criteria were included. Study criteria were the following: (1) age ≥ 18 years, (2) a diagnosis of pulmonary TB, (3) at least one of three sputum smear samples showing acid-fast bacilli (AFB) and sputum culture isolates positive for *M*. *tuberculosis* within 1 month of pulmonary TB diagnosis, (4) availability of DST results, and (5) availability of chest radiograph within 1 month of pulmonary TB diagnosis. Exclusion criteria were (1) no available documentation on the diagnosis and/or management of previous pulmonary TB and/or present pulmonary TB, (2) limited quality of chest radiograph, (3) pregnancy, and (4) mixed infection with non-TB mycobacteria.

Of 558 patients with pulmonary MDR-TB, 145 patients fulfilled the study criteria; 413 patients were excluded as follows: 33 patients had mixed infection with non-TB mycobacteria, 42 patients were < 18 years, 54 patients had no available DST results from the microbiology laboratory records of the central laboratory at the CCIT, 69 patients had no available chest radiographs or had chest radiographs of limited quality, 96 patients had negative sputum AFB smears or sputum showing no growth of *M*. *tuberculosis* upon culture as based on the microbiology laboratory records of the central laboratory at the CCIT, and 119 patients had no available documentation on the diagnosis and management of previous pulmonary TB and/or present pulmonary TB. Thus, 145 patients with pulmonary MDR-TB were recruited into the study as cases; these included 140 (96.6%) patients with previously treated pulmonary TB and 5 (3.4%) patients with newly diagnosed pulmonary TB. Previously treated TB patients were defined as patients with TB who had a prior history of treatment with anti-TB drugs for > 1 month and newly diagnosed TB patients were defined as patients with TB who had never received treatment for TB or patients with TB who had taken any anti-TB drugs for less than 1 month [35].

**Fig 1:**
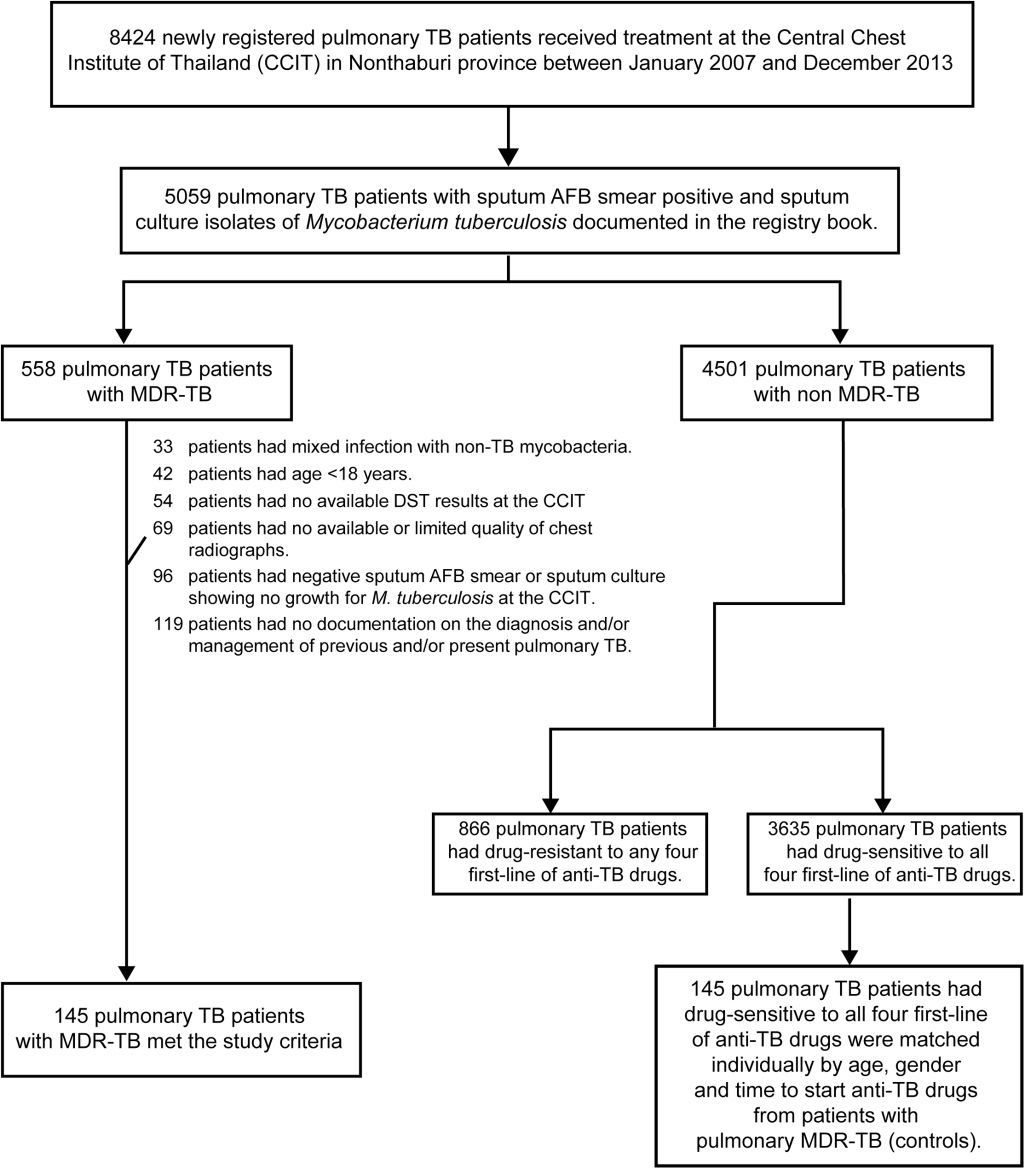
Flow diagram of the study.

Of 4501 patients with pulmonary non-MDR-TB, 866 patients had TB that was drug-resistant to any four first-line anti-TB drugs and 3635 patients had TB that was drug-sensitive to all four first-line anti-TB drugs. Of 3635 patients with pulmonary non-MDR-TB who were drug-sensitive to all four first-line anti-TB drugs, 145 patients from the same period as the cases were recruited into the study as controls. Each of the MDR-TB cases was matched individually with a control in terms of time to initiation of anti-TB drugs (± 2 weeks), age (± 5 years), and gender. Where the medical records, microbiology laboratory records, or chest radiographs of these controls were not available, we selected the next available matched control patient. Controls were thus individually matched to cases, in a ratio of 1:1 (Fig 1). De-identified patient data ensured anonymity before analysis. Demographic information, baseline characteristics, clinical as well as laboratory findings of current TB and management of previous TB, with outcomes, were summarized and recorded in a pre-defined case record form.

### Management and outcomes of patients with pulmonary TB

Patients with smear-positive pulmonary TB were managed following the WHO recommendations, using a category I regimen consisting of INH, RMP, EMB, and PZA for 2 months (2HRZE) in the initiation phase, followed by INH and RMP for 4 months (4HR) in the continuation phase [4]. Patients who received anti-TB drugs other than category I regimen was defined as non-category I regimen. Directly Observed Treatment of Short Course (DOTS) was also implemented during TB management. Three samples of sputum smears were analyzed for AFB at the end of 2, 5, and 6 months of treatment. Treatment outcomes were evaluated after anti-TB treatment. Cure was defined as patients whose sputum was smear-negative for AFB in the last month of treatment, whereas completed treatment was defined as patients who had complete a course of anti-TB drugs, but lacked a result for the sputum smear or culture in the last month of treatment. Patients who remained sputum smear-positive for AFB after 5 months of treatment were defined as those with treatment failure. Defaulted was defined as patients who had interrupted treatment for at least 2 consecutive months [4]. Sputum culture for mycobacteria and DST results with a treatment regimen for MDR-TB was recommended for those with treatment failure. The MDR-TB treatment regimen for patients with pulmonary MDR-TB or those with treatment failure was modified after obtaining DST results. The MDR-TB regimen consisted of at least four anti-TB drugs, including at least three oral anti-TB drugs and one injectable anti-TB drug, according to the WHO recommendations, for at least six months in the initiation phase followed by all oral drugs for at least 12 months in the continuation phase [35]. After the patient had undergone the MDR-TB treatment regimen, three samples of sputum smears and culture were analyzed monthly until sputum culture showed negative results for mycobacteria [35].

### Bacteriologic examinations and identifications

Sputum samples for AFB smear and culture for mycobacteria were performed at the central laboratory of the CCIT, which is one of the reference laboratory centers for mycobacteria in Thailand. Sputum AFB smear tests involved staining using the Ziehl−Neelsen method and the result was reported semi-quantitatively [36]. The results of the AFB smears were interpreted using the WHO 1998 Laboratory Services in the TB Control Grading System, according to which AFB < 1+ was defined as 1–9 AFB per 100 oil immersion fields (OIFs), 1+ as 10–99 AFB per 100 OIFs, 2+ as 1–10 AFB per OIF, and 3+ as > 10 AFB per OIF [36]. The mycobacterium in sputum samples was cultured and DST performed for four anti-TB drugs, including INH, RMP, EMB, and SM using Lowenstein−Jensen media according to the WHO recommendation [36].

### Interpretation of chest radiographic findings

Posterior−anterior chest radiographs within 1 month of pulmonary TB diagnosis were used for interpretation and were obtained using a plain radiography system (Model UD150B-40, Shimadzu Corporation, Kyoto, Japan). The chest radiographs were collected, reviewed, and interpreted in a blinded fashion, in random order, by two independent specialists, including one specialist in pulmonary medicine and one specialist in tropical diseases, neither of whom were the treating physicians and both of whom had more than 10 years’ experience.

**Fig 2:**
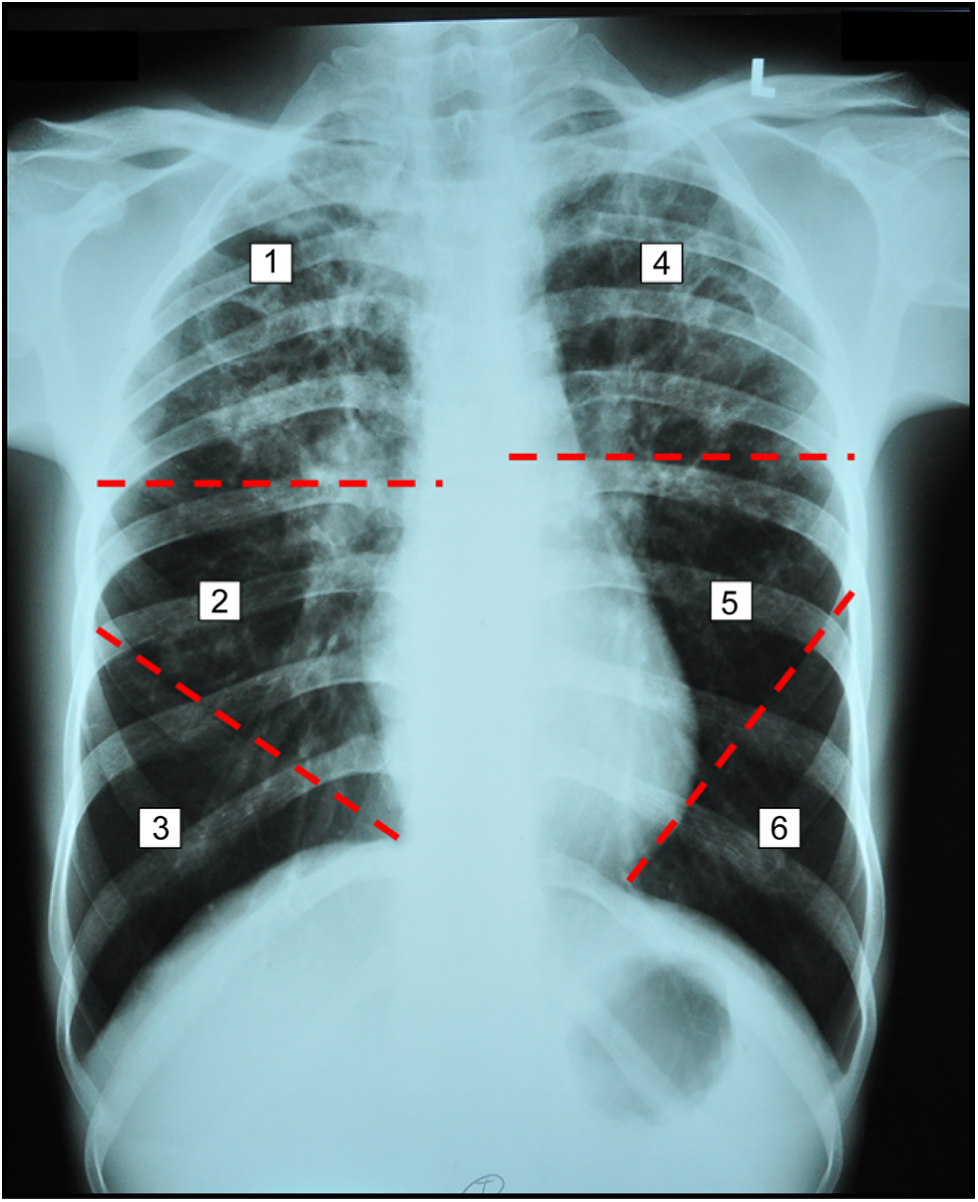
Chest radiograph showing multiple cavities in both upper zones of the six lung zones using as landmark the mid-section of the hilar structure, the second anterior rib below the mid-section of the hilar structure, and the cardiac angle.

The pattern of lung abnormalities, site of lung involvement, and characteristics of lung cavities were defined before interpreting chest radiographs. The pattern of lung abnormalities in pulmonary TB, including reticulo-nodular opacity, reticular opacity, nodular opacity, consolidation, cavities, pleural effusion, atelectasis, and adenopathy were evaluated. For defining the site of lung involvement, the lung was divided into six zones for each patient, as shown in Fig 2. The upper zone was located cephalad to the mid-part of the hilar structure. The lower zone was located caudad to the second anterior rib, below the line of the mid-section of the hilar structure. The middle zone was located between the line of the mid-section of the hilar structure and the line of second anterior ribs below the mid-section of the hilar structure. The characteristics of lung cavities recorded were the maximum diameter of the cavities, the number of cavities, and the site and lung zone involved.

The pattern of lung abnormalities, the extent of lung parenchymal abnormalities, including the site and number of lung zones involved, and the consensus chest radiographic findings were summarized and recorded in a pre-defined case record form for chest radiographic findings. If controversy arose, a third expert repeatedly reviewed the cases based on the interpretation of the previous two specialists. The original two specialists repeated their analysis of the chest radiographs in the same format after 3 months.

### Statistical analysis

All data analyses were performed with SPSS for Windows 18.0 (SPSS Inc. Chicago, IL). Categorical variables were compared with Chi-square analyses or Fisher’s exact test where appropriate, and data for these variables are summarized as number and percentage. The Extended Mantel− Chi-square test for linear trends was used to determine a linear trend of grading of the AFB smear and lung zones of cavities in relation to the occurrence of MDR-TB. Univariate analysis was performed to determine possible factors that could be associated with MDR-TB. All variables with *P* ≤ 0.2 in the univariate logistic regression analysis was included in the stepwise multiple logistic regression analysis, by using the backward selection method for determining independent MDR-TB-associated factors. All tests of significance were two-sided, and *P* < 0.05 was considered statistically significant.

## Results

A total of 290 pulmonary TB patients who received treatment at the CCIT were recruited into the study; 145 were pulmonary TB patients with MDR-TB, defined as cases, and 145 were pulmonary TB patients with drug-sensitive TB, defined as controls (Fig 1). Among the 145 patients with pulmonary MDR-TB, the DST results showed drug-resistance to INH and RMP in all patients, EMB-resistance in 30 (20.7%) patients, and SM resistance in 88 (60.7%) patients.

### Comparison of baseline characteristics and clinical and laboratory findings between patients with MDR-TB and those with drug-sensitive TB

There were no significant differences in the age, gender, residential area, education, and underlying medical illnesses, including diabetes mellitus, cardiovascular diseases, HIV infection, and lung diseases, among patients with MDR-TB and those with drug-sensitive TB. All statistical comparisons for the baseline characteristics of patients with MDR-TB or those with drug-sensitive TB are reported in Table 1.

**Table 1:** Baseline characteristics of 145 patients with pulmonary MDR-TB and 145 patients with drug-sensitive pulmonary TB.

Characteristic(s)	Multidrug-resistant TB		Drug-sensitive TB		Odds ratio (95% CI)	P-value
	n	no. (%)	n	no. (%)		
Age	145		145			0.636
< 40 years		67 (46.2)		62 (42.8)	Reference	
≥40 years		78 (53.8)		83 (57.2)	0.87 (0.55−1.38)	
Gender	145		145			1.000
Female		50 (34.5)		50 (34.5)	Reference	
Male		95 (65.5)		95 (65.5)	1.00 (0.62−1.62)	
Residential area	145		145			1.000
Bangkok		41 (28.3)		42 (29.0)	Reference	
Others		104 (71.7)		103 (71.0)	1.03 (0.62−1.72)	
Education	128		119			0.982
Secondary or above		55 (43.0)		50 (42.0)	Reference	
Illiteracy or primary		73 (57.0)		69 (58.0)	0.96 (0.58−1.59)	
Diabetes mellitus	145		145			0.111
No		116 (80.0)		127 (87.6)	Reference	
Yes		29 (20.0)		18 (12.4)	1.76 (0.93−3.34)	
Cardiovascular diseases	145		145			0.574
No		131 (90.3)		127 (87.6)	Reference	
Yes		14 (9.7)		18 (12.4)	0.75 (0.36−1.58)	
HIV infection	145		145			0.539
No		138 (95.2)		141 (97.2)	Reference	
Yes		7 (4.8)		4 (2.8)	1.79 (0.51−6.24)	
Lung diseases	145		145			1.000
No		140 (96.6)		140 (96.6)	Reference	
Yes		5 (3.4)		5 (3.4)	1.00 (0.28−3.53)	

MDR, multidrug-resistant; TB, tuberculosis; CI, confidence interval.

Of the 145 patients with MDR-TB, 140 (96.6%) patients had prior episodes of pulmonary TB, including 94 (67.1%) patients who had 1 prior episode of pulmonary TB and 46 (32.9%) patients who had ≥ 2 prior episodes of pulmonary TB whereas 5 (3.4%) patients had no prior episode of pulmonary TB. Of 145 patients with drug-sensitive TB, 123 (84.8%) patients had no prior episode of pulmonary TB, whereas 22 (15.2%) patients had prior episodes of pulmonary TB, including 20 (90.9%) patients who had 1 prior episode of pulmonary TB and only 2 (9.1%) patients who had ≥ 2 prior episodes of pulmonary TB. The proportion of patients with MDR-TB who had ≥ 2 prior episodes of pulmonary TB was significantly higher than those with drug-sensitive TB (46 [31.7%] vs. 2 (1.4%) patients, *P* < 0.001; Table 2).

**Table 2:** Clinical and laboratory findings of 145 patients with pulmonary MDR-TB and 145 patients with drug-sensitive pulmonary TB.

Characteristic(s)	Multidrug-resistant TB		Drug-sensitive TB		Odds ratio (95%CI)	P-value
	n	no. (%)	n	no. (%)		
*Previous pulmonary TB status*						
Number of prior TB	145		145			< 0.001
≤ 1 episode		99 (68.3)		143 (98.6)	Reference	
≥ 2 episodes		46 (31.7)		2 (1.4)	33.22 (7.88−140.04)	
Prior anti-TB regimen	140		22			0.012
Cat I regimen		93 (66.4)		21 (95.5)	Reference	
Non-Cat I regimen		47 (33.6)		1 (4.5)	10.61 (1.38−81.34)	
Known outcome of TB	136		18			0.036
Cure or complete		28 (20.6)		8 (44.4)	Reference	
Failure or default		108 (79.4)		10 (55.6)	3.09 (1.12−8.54)	
*Presentation of present pulmonary TB*						
Duration of illness	135		143			< 0.001
≤ 60 days		55 (40.7)		104 (72.7)	Reference	
> 60 days		80 (59.3)		39 (27.3)	3.88 (2.34−6.42)	
Body mass index	119		120			0.052
≥ 18.0 kg/m^2^		69 (58.0)		85 (70.8)	Reference	
< 18.0 kg/m^2^		50 (42.0)		35 (29.2)	1.76 (0.99−3.12)	
Cough	145		145			< 0.001
Yes		117 (80.7)		139 (95.9)	Reference	
No		28 (19.3)		6 (4.1)	5.54 (2.22−13.85)	
Fever	145		145			< 0.001
Yes		49 (33.8)		86 (59.3)	Reference	
No		96 (66.2)		59 (40.7)	2.86 (1.77−4.60)	
Hemoptysis	145		145			0.027
Yes		13 (9.0)		27 (18.6)	Reference	
No		132 (91.0)		118 (81.4)	2.32 (1.15−4.71)	
*Laboratory findings*						
Sputum AFB smear	145		145			< 0.001
1+		11 (7.6)		41 (28.3)	Reference	
2+		28 (19.3)		81 (55.9)	1.29	
3+		106 (73.1)		23 (15.9)	17.18	
Abnormal chest radiological finding(s)						
Abnormal findings	145		145			< 0.001
1 pattern		39 (26.9)		103 (71.0)	Reference	
≥ 2 patterns		106 (73.1)		42 (29.0)	6.66 (3.99−11.14)	
Reticulo-nodular	145		145			0.900
No		46 (31.7)		48 (33.1)	Reference	
Yes		99 (68.3)		97 (66.9)	1.06 (0.65−1.74)	
Cavities	145		145			< 0.001
No		53 (36.6)		111 (76.6)	Reference	
Yes		92 (63.4)		34 (23.4)	5.67 (3.40−9.45)	
Consolidation	145		145			0.203
No		95 (65.5)		106 (73.1)	Reference	
Yes		50 (34.5)		39 (26.9)	1.43 (0.87−2.36)	
Pleural effusion	145		145			0.002
No		110 (75.9)		131 (90.3)	Reference	
Yes		35 (24.1)		14 (9.7)	2.98 (1.52−5.82)	
Atelectasis	145		145			0.005
No		124 (85.5)		139 (95.9)	Reference	
Yes		21 (14.5)		6 (4.1)	3.92 (1.53−10.03)	
Nodules	145		145			0.070
No		130 (89.7)		139 (95.9)	Reference	
Yes		15 (10.3)		6 (4.1)	2.67 (0.94−7.98)	
Reticular	145		145			1.000
No		140 (96.6)		140 (96.6)	Reference	
Yes		5 (3.4)		5 (3.4)	1.00 (0.28−3.53)	
Adenopathy	145		145			0.748
No		141 (97.2)		139 (95.9)	Reference	
Yes		4 (2.8)		6 (4.1)	0.66 (0.18−2.38)	

MDR, multidrug-resistant; TB, tuberculosis; OR, odds ratio; CI, confidence interval; Cat, category; AFB, acid-fast bacilli.

Of the 140 MDR-TB patients with prior episodes of pulmonary TB, 93 (66.4%) patients received treatment with a category I anti-TB regimen and 47 (33.6%) patients received treatment with a non-category I regimen. Of the 22 drug-sensitive TB patients with prior episodes of pulmonary TB, 21 (95.5%) patients received treatment with a category I anti-TB regimen and only 1 (4.5%) patient received treatment with a non-category I regimen. The proportion of patients with MDR-TB who received a non-category I regimen was significantly higher than those with drug-sensitive TB (47/140 [33.6%] patients vs. 1/22 [4.5%] patients, *P* = 0.012; Table 2).

Treatment outcomes of a prior anti-TB regimen among patients with MDR-TB included treatment failure (88 patients, 62.9%) followed by cure or completed treatment (28 patients, 20.6%), default (20 patients, 14.3%), and referral to other hospitals for treatment (4 patients, 2.9%). Among the patients with drug-sensitive TB, treatment outcomes of a prior anti-TB regimen included default (10 patients, 45.5%) followed by cure or completed treatment (8 patients, 36.4%), referral to other hospitals for treatment (4 patients, 18.2%); there were no treatment failures. The proportion of treatment failures or default outcomes among patients with MDR-TB was significantly higher than among those with drug-sensitive TB (108 [79.4%] vs. 10 [55.6%] patients, *P* = 0.036; Table 2).

In terms of the presentation of current pulmonary TB, the proportion of patients with a duration of illness > 60 days was significantly higher among patients with MDR-TB than those with drug-sensitive TB (80/135 [59.3%] patients vs. 39/143 [27.3%] patients, *P* < 0.001]. Cough was the most common form of presentation of patients with pulmonary TB, but the proportion of patients without cough was significantly higher among patients with MDR-TB than among those with drug-sensitive TB (28 [19.3%] patients vs. 6 [4.1%] patients, *P* < 0.001). In addition, patients with MDR-TB were more likely to have no fever (96 [66.2%] patients vs. 59 [40.7%] patients, *P* < 0.001) and no hemoptysis (132 [91.0%] patients vs. 118 [81.4%] patients, *P* = 0.027; Table 2).

Regarding laboratory findings among patients with pulmonary TB, there was strong evidence of a linear trend in terms of the proportion of patients with MDR-TB in relation to sputum AFB smear grading (Chi-square for linear trend = 79.26, *P* < 0.001; Table 2). In order to assess the chest radiographic interpretation, the kappa statistics for intra- and inter-observer agreement were performed as shown in Table 3. The overall intra- and inter-observer agreement were good to excellent between the two specialists, except that the maximum diameter of cavities showed moderate agreement in the second round of chest radiograph interpretation. According to the chest radiological findings, the number of abnormal radiological findings of ≥ 2 patterns was more commonly observed in patients with MDR-TB than in those with drug-sensitive TB (106 [73.1%] patients vs. 42 [29.0%] patients, *P* < 0.001). Patients with MDR-TB were more likely to present with (1) cavities (92 [63.4%] patients vs. 34 [23.4%] patients, *P* < 0.001), (2) pleural effusion (35 [24.1%] patients vs. 14 [9.7%] patients, *P* = 0.002), and (3) atelectasis (21 [14.5%] patients vs. 6 [4.1%] patients, *P* = 0.005) than those with drug-sensitive TB (Table 2).

**Table 3:** Kappa coefficient (95% confidence interval) for the intra- and inter-observer agreement in the interpretation of chest radiograph at the first and the second readings.

	Specialist in Pulmonary Medicine		Specialist in Tropical Diseases		Specialists in Pulmonary Medicine/ Tropical Diseases		Specialists in Pulmonary Medicine/ Tropical Diseases	
	R1 and R2		R1 and R2		R1 and R1		R2 and R2	
	Kappa	95% CI	Kappa	95% CI	Kappa	95% CI	Kappa	95% CI
Reticulo-nodular	0.90	0.85−0.95	0.90	0.84−0.95	0.92	0.88−0.97	0.87	0.81−0.93
Reticular	1.00	-	0.77	0.58−0.97	0.84	0.65−1.02	0.83	0.66−0.99
Nodules	0.95	0.88−1.02	0.90	0.80−1.00	0.92	0.83−1.01	0.87	0.76−0.98
Cavities	0.97	0.94−0.99	0.96	0.92−1.06	0.98	0.96−1.00	0.96	0.93−1.00
Max diameter ≥ 30 mm	0.83	0.74−0.93	0.85	0.76−0.94	0.68	0.55−0.80	0.57	0.42−0.72
Number of cavities ≥ 3	0.93	0.86−1.00	0.84	0.73−0.94	0.95	0.89−1.01	0.86	0.76−0.95
Bilateral involvement	0.92	0.84−0.99	0.93	0.87−1.00	0.97	0.92−1.01	0.92	0.85−0.99
Lung zone ≥ 2	0.90	0.83−0.98	0.94	0.87−1.00	0.97	0.92−1.01	0.90	0.83−0.98
Consolidation	0.92	0.86−0.97	0.88	0.82−0.94	0.91	0.86−0.96	0.94	0.84−0.96
Effusion	0.98	0.94−1.01	0.89	0.82−0.96	0.93	0.87−0.98	0.92	0.86−0.98
Atelectasis	0.93	0.85−1.01	0.84	0.72−0.96	0.87	0.76−0.97	0.92	0.83−1.00
Adenopathy	0.95	0.84−1.05	0.85	0.69−1.02	0.95	0.84−1.05	0.95	0.85−1.04

R1, first reading; R2, second reading; CI, confidence interval; Max, maximum.

### Univariate and multivariate analysis for the occurrence of pulmonary MDR-TB

We used univariate logistic regression analysis to ascertain which of the baseline characteristics, previous history of TB, clinical, and laboratory findings were associated with the occurrence of pulmonary MDR-TB. All factors that were significantly associated with pulmonary MDR-TB were included in the univariate logistic regression analysis. We identified the following factors associated with pulmonary MDR-TB: (1) ≥ 2 episodes of prior pulmonary TB, (2) duration of illness > 60 days, (3) having a sputum AFB smear grading 2+ or 3+, (4) presence of cavities, (5) presence of pleural effusion, and (6) presence of atelectasis (Table 4).

**Table 4:** Univariate analysis for associated clinical and laboratory findings for the occurrence of pulmonary MDR-TB.

Characteristic(s)	Univariate logistic regression analysis			P-value
	n	Odds ratio	95% CI	
*Clinical findings*				
Number of prior TB	290			< 0.001
≤ 1 episode		1.00	Reference	
≥ 2 episodes		33.22	7.88−140.04	
Duration of illness	278			< 0.001
≤ 60 days		1.00	Reference	
> 60 days		3.88	2.34−6.42	
*Laboratory findings*				
Sputum AFB positive	290			< 0.001
1+		1.00	Reference	
2+		1.29	0.58−2.84	0.53
3+		17.18	7.69−38.38	< 0.001
Abnormal chest radiological finding(s)				
Cavities	290			< 0.001
No		1.00	Reference	
Yes		5.67	3.40−9.45	
Pleural effusion	290			0.001
No		1.00	Reference	
Yes		2.98	1.52−5.82	
Atelectasis	290			0.004
No		1.00	Reference	
Yes		3.92	1.53−10.03	

MDR, multidrug-resistant; TB, tuberculosis; CI, confidence interval; AFB, acid fast bacilli.

After including all parameters with a *P* ≤ 0.2 in the univariate logistic regression analysis in a stepwise multiple logistic regression analysis, using backward selection, we found the following risk factors to be independently associated with the occurrence of pulmonary MDR-TB: (1) ≥ 2 episodes of prior pulmonary TB (OR [95% CI] = 39.72 [7.86−200.66], *P* < 0.001), (2) duration of illness > 60 days (OR [95% CI] = 3.08 [1.52−6.22], *P* = 0.002), (3) having a sputum AFB smear score of 3+ (OR [95% CI] = 13.09 [4.64−36.91], *P* < 0.001), and (4) the presence of cavities (OR [95% CI] = 3.82 [1.89−7.73], *P* < 0.001), and (5) the presence of pleural effusion (OR [95% CI] = 2.75 [1.06−7.16], *P* = 0.038). These findings are shown in Table 5.

**Table 5:** Multivariate logistic regression analysis for associated clinical and laboratory findings for the occurrence of pulmonary MDR-TB.

Characteristic(s)	Multivariate logistic regression analysis			P-value
	n	Odds ratio	95% CI	
*Clinical findings*				
Number of prior TB	278			< 0.001
≤ 1 episode		1.00	Reference	
≥ 2 episodes		39.72	7.86−200.66	
Duration of illness	278			0.002
≤ 60 days		1.00	Reference	
> 60 days		3.08	1.52−6.22	
*Laboratory findings*				
Sputum AFB positive	278			< 0.001
1+		1.00	Reference	
2+		1.02	0.36−2.94	0.968
3+		13.09	4.64−36.91	< 0.001
Abnormal chest radiological finding(s)				
Cavities	278			< 0.001
No		1.00	Reference	
Yes		3.82	1.89−7.73	
Pleural effusion	278			0.038
No		1.00	Reference	
Yes		2.75	1.06−7.16	

Predictors entering the model: number of prior TB; duration of illness; sputum AFB positive; cavities; pleural effusion; atelectasis. MDR, multidrug-resistant; TB, tuberculosis; CI, confidence interval; AFB, acid fast bacilli.

### Comparison of cavity pattern between patients with MDR-TB and those with drug-sensitive TB

The cavity patterns noted by chest radiography are shown in Table 6. There were 92 (63.4%) patients with pulmonary MDR-TB and 34 (23.4%) patients with drug-sensitive pulmonary TB who had cavities upon chest radiography. Patients with pulmonary MDR-TB had a significantly higher proportion of cavities with a maximum diameter ≥ 30 mm (77 [83.7%] patients vs. 5 [14.7%] patients, *P* < 0.001), ≥ 3 cavities (42 [45.7%] patients vs. 4 [11.8%] patients, *P* = 0.001), bilateral involvement (48 [52.2%] patients vs. 5 [14.7%] patients, *P* < 0.001), and ≥ 2 lung zones involved (55 [59.8%] patients vs. 8 [23.5%] patients, *P* = 0.001) than those with drug-sensitive pulmonary TB. In addition, there was strong evidence of a linear trend in the proportion of patients with pulmonary MDR-TB in relation to the number of lung zones involved (Chi-square for linear trend = 13.44, *P* = 0.001).

**Table 6:** Chest radiological findings of cavity patterns among 92 patients with pulmonary MDR-TB and 34 patients with drug-sensitive pulmonary TB.

Characteristics	Multidrug-resistant TB	Drug-sensitive TB	Odds ratio (95%CI)	P-value
	no. (%)	no. (%)		
Max diameter (mm)				< 0.001
< 30	15 (16.3)	29 (85.3)	Reference	
≥ 30	77 (83.7)	5 (14.7)	29.77 (9.92−89.31)	
Number of cavities				0.001
< 3	50 (54.3)	30 (88.2)	Reference	
≥ 3	42 (45.7)	4 (11.8)	6.30 (2.05−19.33)	
Site of cavities				< 0.001
Unilateral	44 (47.8)	29 (85.3)	Reference	
Bilateral	48 (52.2)	5 (14.7)	6.33 (2.25−17.78)	
Lung zone involvement				0.001
1	37 (40.2)	26 (76.5)	Reference	
2	35 (38.0)	7 (20.6)	3.51	
≥ 3	20 (21.7)	1 (2.9)	14.05	

MDR, multidrug-resistant; TB, tuberculosis; OR, odds ratio; CI, confidence interval; Max, maximum.

## Discussion

In our study, patients with pulmonary MDR-TB had similar underlying medical illnesses as patients with drug-sensitive pulmonary TB. The number of patients with HIV infection was also similar among patients with pulmonary MDR-TB and patients with drug-sensitive pulmonary TB, which supported previous reports showing that HIV infection was not one of the risk factors for the development of MDR-TB [7,8]. A number of previous reports showed that a history of previously treated TB was one of the risk factors for MDR-TB [9–14, 17–23]. In our study, patients with ≥ 2 previous episodes of pulmonary TB had a risk for development of pulmonary MDR-TB (OR 39.72). Our study also showed that patients with previous pulmonary TB episodes who were treated with a non-category I regimen and where the treatment outcome of previous pulmonary TB was treatment failure or default were more likely to be now have pulmonary MDR-TB than drug-sensitive pulmonary TB. Thus, the management of prior episodes of pulmonary TB is an important factor in the development of pulmonary MDR-TB.

A previous study showed that the majority of the patients with pulmonary TB (75.8%) presented with cough, one-half of the patients (50.6%) presented with fever, and approximately one-fourth of the patients (23.8%) presented with hemoptysis [37]. Our study demonstrated a significantly higher proportion of patients with pulmonary MDR-TB who presented with no cough, no fever, and no hemoptysis than did patients with drug-sensitive pulmonary TB. Additionally, presentation with illness of long duration (> 60 days, OR 3.08) was a risk factor for the development of MDR-TB. It is likely that these patients received partial treatment for TB before receiving treatment at the CCIT or that a less virulent strain became dominant after the development of drug-resistance [38].

A previous study also showed that patients with MDR-TB had a significantly higher sputum smear-positivity rate than those with mono-resistant or drug-sensitive TB (MDR-TB in 80.0% vs. mono-resistant in 50.0% vs. drug-sensitive TB in 53.3%, *P* < 0.001) [39]. In addition, a higher rate of sputum smear-positivity was associated with advanced chest radiographic findings in a previous report [40]. However, our study showed that a sputum AFB smear-positive score of 3+ (OR 13.09) and the presence of cavities (OR 3.82) upon chest radiography were independent risk factors for the development of MDR-TB. Additionally, the characteristics of cavities, including having a maximum diameter ≥ 30 mm, number of cavities ≥ 3, bilateral involvement, and the presence of cavities in ≥ 2 lung zones, were associated with the development of MDR-TB. Our findings were similar to previous reports from Korea showing that cavity formation, particularly the presence of multiple cavities (>3 cavities), was associated with the development of MDR-TB [30,41].

In our study, pleural effusion was another risk factor for the development of MDR-TB (OR 2.75). The occurrence of pleural effusion in patients with drug-sensitive pulmonary TB was 8.6% and increased to 24.3% among patients with pulmonary MDR-TB. These findings are probably due to the entry of more mycobacteria into the pleural space due to the high burden of organisms among patients with MDR-TB, leading to stimulation of the delayed hypersensitivity reaction to mycobacterial antigens [42,43] or the occurrence of an immunological response after initiation of anti-TB drugs for approximately 2 months [44]. A previous study showed that pleural TB was the second most common extrapulmonary form of TB; in drug-resistant TB, this form of TB accounted for approximately 4% of TB cases [43].

In our study, there was a high rate of drug-resistance to SM (60.7%) and a moderate rate of drug-resistance to EMB (20.7%) among patients with pulmonary MDR-TB. According to the WHO recommendation, SM should not be used as a standard treatment regimen for MDR-TB due to the high SM-resistance rate in MDR-TB isolates; EMB may be considered for treatment of MDR-TB if the DST results show drug susceptibility [35].

The treatment of MDR-TB requires more complex therapy with longer treatment duration (18−24 months); thus, adverse drug events occur more frequently compared to the treatment regimen for non-MDR-TB [35,45]. In terms of treatment of MDR-TB in Thailand, the clinical practice has been to select a regimen of at least four susceptible anti-TB drugs, including 1 injectable anti-TB drug and at least 3 oral anti-TB drugs, as per the WHO recommendations [35]. The common anti-TB drugs used for treatment of MDR-TB in Thailand include intramuscular kanamycin, with oral levofloxacin, ethionamide, cycloserine, and p-aminosalicylic acid during the initiation phase (for 6−8 months), followed by all oral drugs after sputum culture has been negative for 18 months, in the continuation phase. However, pyrazinamide is not recommended in the standard empiric regimen for MDR-TB due to the high pyrazinamide-resistance rate in MDR-TB isolates (49.0%) in Thailand [46].

Our study had some limitations. (1) The majority of patients in the control group were patients who did not have a history of previously treated pulmonary TB, as most of the patients with previously treated pulmonary TB developed drug-resistance after receiving anti-TB drugs. (2) Patients with sputum AFB-positive smears were recruited into our study, which may have induced a sampling bias. The reason for recruiting such patients was to avoid the possibility of inappropriate collection of sputum samples or improperly prepared sputum AFB smears, which may have affected the smear test. (3) Availability of certain data, such as the mode of TB contact, and DST for second-line drugs, was limited due to the retrospective nature of the study. (4) Our study is a retrospective matched case−control study. In Thailand, the occurrence of pulmonary MDR-TB is a rare condition and the long duration of the study is essential for including sufficient numbers of patients into the study. Therefore, changes in the incidence of newly diagnosed and previously treated MDR-TB over time might influence the risk factors for MDR-TB.

In conclusion, the risk factors for MDR-TB among patients with pulmonary TB included ≥ 2 episodes of prior pulmonary TB, duration of illness > 60 days, a sputum AFB smear score of 3+, and the presence of cavities or pleural effusion on chest radiographs. These clinical factors and chest radiographic findings associated with MDR-TB should prompt physicians to perform DST sooner, and to provide proper management of cases, which may reduce the number of patients with MDR-TB in future.
